# Co-authorship network analysis in health research: method and potential use

**DOI:** 10.1186/s12961-016-0104-5

**Published:** 2016-04-30

**Authors:** Bruna de Paula Fonseca e Fonseca, Ricardo Barros Sampaio, Marcus Vinicius de Araújo Fonseca, Fabio Zicker

**Affiliations:** Alberto Luiz Coimbra Institute for Graduate Studies and Research in Engineering (COPPE), Federal University of Rio de Janeiro (UFRJ), Av Horacio Macedo 2030, Center of Technology, room G207, Rio de Janeiro, 21941-914 Brazil; Center for Technological Development in Health (CDTS), Oswaldo Cruz Foundation (Fiocruz), Av Brasil 4036, 8th floor, room 814, Rio de Janeiro, 21040-361 Brazil; Diretoria Regional de Brasilia (DIREB), Oswaldo Cruz Foundation (Fiocruz), Av L3 Norte, s/n, Campus Universitário Darcy Ribeiro, Gleba A, Brasília, 70910-900 Brazil; University of Brasília, Campus Universitário Darcy Ribeiro, Edifício da Biblioteca Central, Entrada Leste, Brasília, 70910-900 Brazil

## Abstract

Scientific collaboration networks are a hallmark of contemporary academic research. Researchers are no longer independent players, but members of teams that bring together complementary skills and multidisciplinary approaches around common goals. Social network analysis and co-authorship networks are increasingly used as powerful tools to assess collaboration trends and to identify leading scientists and organizations. The analysis reveals the social structure of the networks by identifying actors and their connections. This article reviews the method and potential applications of co-authorship network analysis in health. The basic steps for conducting co-authorship studies in health research are described and common network metrics are presented. The application of the method is exemplified by an overview of the global research network for Chikungunya virus vaccines.

## Background

Scientific collaborative networks are a hallmark of contemporary academic research. Scientists are no longer independent players, but members of scientific cooperation networks looking for solutions to social, political, economic and technological problems, which, usually, require multidisciplinary approaches [1]. When collaborating, researchers can establish communication networks, share ideas, resources and information, generate and deliver new knowledge, and ultimately create innovations, reducing the cost and increasing the productivity of research [2].

Collaborative networks are particularly relevant in health innovation because of its complexity, involving multiple stakeholders and increasingly dependent on interdisciplinary research [3]. Given the diverse disciplines that cut across health innovations, knowledge networks are needed to address such complex areas [4, 5]. Health innovation networks are proposed as efficient strategies to help developing countries to address neglected tropical disease challenges [6]. Open innovation networks between industry and academia are critical to accelerate the development of appropriate products for the health systems [7].

Recent studies have proposed the use of social network analysis (SNA) to: (1) support the evaluation of cross-disciplinary research programs [8]; (2) develop strategic public policy planning [9]; and (3) strengthen innovation management in public health systems [10]. Other applications of SNA include support to organizational competitive intelligence [11] and communication management of networks for the health innovation system [12].

This article reviews the SNA methodology, as applied to scientific co-authorship in health research, to assess collaboration trends, identify leading investigators and organizations, and explain the influence of external factors in research collaboration and scientific productivity. As an example, we have analyzed the global network of research organizations working with the development of vaccines against the Chikungunya virus.

## Review

### Social network analysis

A social network can be defined as a finite set of actors (or nodes) and the relationships (or links) between them. It is the presence of this relational information that characterizes a social network, being a critical resource and a prerequisite for its definition [13, 14]. SNA is a theoretical perspective and a set of techniques used to understand and quantitatively measure these relationships [14]. Its main feature is the emphasis, not on the characteristics or attributes of the actors, but on the connections between them [13]. By quantifying the social structure of a network – the set of nodes and their connections – it is possible to identify the most important nodes, the formation of groups and the flow of tangible and intangible resources, among other information [13, 15].

The choice of nodes to be analyzed depends on the context in which a given set of data is inserted. These nodes can be individuals, groups, organizations and even whole countries. In SNA, each relationship defines a different type of network. A connection between the nodes can be the shared authorship of a paper, the participation on the same project or the organization of a scientific event. From the perspective of SNA, relations are not properties of nodes, but of whole systems.

### Co-authorship networks

Scientific collaboration can be defined as the interaction that takes place within a social context between two or more scientists, which facilitates the sharing of meaning and fulfillment of tasks in relation to a mutually shared goal [1]. Scientists are driven to collaborate due to the opportunity to discover new knowledge, the increasing specialization within science, the complexity of infrastructure required as well as the need to combine different types of knowledge and skills to address complex health problems [1, 16]. Scientific collaboration can also help broaden the scope of a research project and foster innovation as it provides access to different disciplines [17].

Co-authorship analysis in science and technology (S&T) partnerships provides a vision of cooperation patterns between individuals and organizations [18–20]. The co-authorship of a technical document is an official statement of the involvement of two or more authors or organizations [20]. Despite the debate about its meaning and interpretation [17, 21], co-authorship analysis is still widely used to understand and assess scientific collaboration patterns.

In co-authorship networks, nodes represent authors, organizations or countries, which are connected when they share the authorship of a paper [20].

### Brief review of the applications of co-authorship analysis in health research

The analysis of co-authorship networks in health research can be used in different contexts. Morel et al. [9] suggested that co-authorship analysis could provide relevant scientific information for strategic planning of health organizations. The authors analyzed the Brazilian scientific co-authorship networks in six neglected tropical diseases (dengue, Chagas disease, leishmaniasis, leprosy, malaria and tuberculosis). Through SNA, they were able to identify key leading organizations that could act as scientific bridges in the scientific community, the most active research groups, the public health approach to these diseases and other relevant information that could contribute to the management of control programs.

Another application was the evaluation of the relationship between scientific productivity and health technological development. The analysis of publication and patent networks in tuberculosis highlighted the active role of the academic area, but a weak engagement of industry in Brazil [10]. The authors recommended the joint analysis of scientific publications and patents in developing countries, where the same actors are usually involved in both research and technological innovation.

González-Alcaide et al. [22] analyzed global research collaboration in leishmaniasis. Using SNA, the authors were able to characterize the collaboration profile, identify key researchers and countries that had the greatest role in the network, and observe the cooperation trend and the development of research groups over time. According to the authors, SNA allows better understanding of the markedly cooperative organizational and social context in which scientific knowledge is generated.

Reviewing over 3000 research articles on health management indexed on the Web of Science database over a period of 13 years, Zhang et al. [23] showed that, despite the growing collaborative behaviour in recent years, cooperation between countries is still incipient and should be encouraged in order to internationalize and promote the progress of research in the area. The authors were able to identify the leading academic researchers in the field and research organizations that played an essential role in disseminating information and control of health management resources.

Naranjo-Estupiñán et al. [24], reviewing co-authorship networks in public health in Colombia, showed that the scope of collaboration in epidemiology is more restrictive than in social sciences, which has a broader engagement of collaborators. The analysis allowed the identification of public health disciplines with poor collaboration.

A study of scientific collaborative networks in biotechnology in the north-eastern region of Brazil [25], showed that collaboration was primarily intra-institutional, with limited diversity and marginal representation of the private sector, not reflecting the expected role of biotechnology as one of the pillars to drive science, technology and innovation in the region.

The review by Robinson-García et al. [26] of inter-organizational relationship of Spanish universities in healthcare allowed the identification of leading universities, as well as the potential collaboration according to their publication profile.

Bender at al. [27] identified and mapped neglected tropical disease research with an affiliation to Germany. The authors acknowledged the strong collaborations between German organizations and partners abroad, but limited collaboration with low- and middle-income countries, highlighting the need of engaging in scientific capacity building efforts. Through the analysis of individual researcher networks, academic talents could be identified.

Table 1 summarizes the potential applications of co-authorship network analysis as exemplified before. Six main objectives and key potential indicators were identified in the literature. Co-authorship analysis allowed assessment of the productivity of research programs, assessment of the relationship between scientific and technological development, mapping of priority thematic areas, evaluation of the regional contribution to knowledge generation, assessment of inter-organizational networks, and assessment of international collaboration.

**Table 1:** Main applications of social network analysis and co-authorship networks in health research

Objective	Target network	Key indicators	References
Assess the extent of collaboration within research programs	Co-authorship in target fields of the research programs	- Changes in network structure before and after the program	Morel et al., 2009 [9]
		- Central organizations and researchers	
Assess the relationship between scientific and technological development	Co-authorship in specific themes in parallel with patent co-inventorship networks	- Central authors and inventors and their relationships	Vasconcellos & Morel, 2012 [10]
		- Differences in the structural properties of both networks	
Map priority thematic areas	Co-authorship in priority themes of public health interest	- Changes in the network structure over time	González-Alcaide et al., 2013 [22] Zhang et al., 2013 [23]
		- Central organizations and researchers	
		- Formation of research groups	
Evaluate the regional contribution to knowledge generation	Regional co-authorship in areas of interest	- Regional collaboration patterns of organizations and researchers	Naranjo-Estupiñán et al., 2014 [24] Costa et al., 2013 [25]
		- Central organizations and researchers	
		- Frequent partners	
Assess inter-organizational networks	Co-authorship of science and technology organizations	- Collaboration patterns (type and frequency of cooperation)	Robinson-García et al., 2013 [26]
Assess international collaboration	Co-authorship between countries	- Scientific collaboration between countries	Bender et al., 2015 [27]
		- Frequent partners	

### Method description

The three main steps of co-authorship analysis are (1) retrieval of scientific publications; (2) standardization of entries for authors and organizations; (3) network visualization and calculation of metrics; and (4) interpretation of results.

#### Data retrieval

Publication records are collected from structured bibliographic databases, to allow systematic cleaning and standardization of data. Ideally, these databases should:

1. Cover a large number of academic journals and have high representation of health-related journals;
2. Provide information on the affiliations of the authors, allowing the construction of organizational networks;
3. Allow the exportation of data in text format compatible with bibliometric analysis software;
4. Provide the full name of the authors in most publications.

In SNA studies, the correct spelling of authors’ names is critical for accurate and reliable links between them and consequently for the entire network. The same author can have different names on records resulting from abbreviations, omissions, name changes, aliases and spelling errors, while different authors can have the same name (homonyms) [28]. These cases can generate errors, causing links to be falsely aggregated or disaggregated. Large differences were found in metrics between co-authorship networks comparing full names, short names or the surname followed by the first initial [29].

The choice of the most appropriate database will depend on the subject of study and on the type of network investigated. Some of them do not disclose the affiliations of all authors of a paper, which hinders the analysis of organizations or country networks. Others cover a wide range of journals, but lack the information about the full name of authors, which can make the process of standardization more difficult.

The objective of the analysis and questions to be answered will guide the lag-time of study. In general, there are two main approaches. The first is based on the premise that by sharing the authorship of a scientific paper, authors must have been collaborating for some time, during which exchange of information took place more intensively [30]. A period of up to 5 years has been used to assess the structure of current cooperation [31–33].

A second approach is cumulative networking over an extended period of analysis. This approach is based on the assumption that social links between authors persist over time, even after the end of a formal collaboration [34]. These cumulative networks are an indication of the growing social structure that potentially functions as a network through which relevant knowledge related to innovation can remain [35].

Publications that have a very large number of authors should be evaluated carefully as, in some cases, they represent just independent contributions of data to joint efforts, involving limited intellectual interactions [36].

#### Standardization and cleaning the data

The step of standardizing and cleaning the retrieved data can be done manually or using specific software depending on the volume of the data and/or availability of software. The objective is to consolidate names of a particular author or organization in order to ensure the correct acknowledgement of their scientific production.

After the cleaning process, individual and organizational co-authorship should be formatted into specific adjacency matrices, edge lists or adjacency lists, in order to map the relationships between the nodes. In matrices, the name of all nodes in the network are entered in both the rows and columns. When a given pair of nodes share the authorship of a paper, the number 1 is placed at the intersection between the two; otherwise, the number 0 is placed at the intersection. If collaboration occurs more than once, the number will be equal to the total of papers co-authored. An edge list is a two-column list of all the node pairs that are connected. In an adjacency list, the whole set of connections of a single node is listed, beginning with the source node and ending with the target nodes. Lists are most useful for representing networks with few links. As co-authorship requires reciprocal cooperation among the participants, all connections are considered to be non-directional.

These data is then imported into a software that allows not only the visualization of networks but also the statistical analysis of the data set, such as Gephi [37], Ucinet [38] and Pajek [39], among others.

#### Network assembly, metrics and visualization

The statistical analysis in co-authorship networks includes quantitative metrics that may reflect the properties of the network as a whole or of its individual nodes. The choice of which metric to use depends upon the context and subjects of the analysis.

The metrics at the network level provide information on its overall structure and properties. Common measurements are (1) the number of nodes and links; (2) density; (3) centralization; and (4) community structure of the network. The number of nodes and links represent the network size. Density is a metric to measure the connectivity within the network and is defined as the percentage of the number of existing links in relation to the maximum number of possible links in a given network [13]. Centralization refers to the degree to which links are concentrated in one or a few nodes in the network, being useful to evaluate if there are ‘dominant’ nodes in the network. The community structure reflects the division of a network into groups or modules whose internal connections are dense and external connections are sparse [40].

Metrics at the individual level describe the importance of a node relative to all other nodes in a given network, taking into account the different ways in which it interacts and communicates with the rest of the network. Centrality measures are the most used in SNA to identify the nodes that have strategic significance in the network. The ‘degree centrality’ can be defined as the number of links that a node has with other nodes. The more relational ties a node has, the more power or prestige it has in a network. The ‘betweeness centrality’ is based on the extent to which a particular node lies between other pairs of nodes in a network, connecting them. Nodes that are often on the shortest path between other nodes are deemed ‘central’ because they control the flow of information in the network by connecting different groups. ‘Closeness centrality’ is based upon the degree to which a node is close to all other nodes in the network. A node is viewed as central to the extent that it does not depend on others as intermediaries of information [41].

Visualization is an important component of SNA. It gives meaning to the analysis and both complement each other. Each network actor (individual, institutional, etc.) is usually displayed as a circle, and its size and/or color can reflect one or more of its characteristics, such as centrality, nationality, gender, etc. Network drawing can be improved for better visibility using spring or force-based algorithms [14].

#### Interpretation of results

The interpretation of the visual display and metrics results is oriented according to key research questions, context and information needed. It has the potential to describe the overall structure of the network, the main actors and their different roles, the impact of the network on different contexts and the factors that may have influenced its configuration and evolution. Such information can be used to guide funding strategies, for strengthening and developing new partnerships, identifying knowledge gaps, evaluating national, regional and international collaboration, mapping priority areas, and as a benchmark to evaluate research programs.

## Example: global research network on vaccines against the Chikungunya virus

In order to illustrate the application of co-authorship network analysis in a research area relevant to public health, we have analyzed the global network of research organizations working in the development of vaccines against the Chikungunya virus (CHIKV).

The mosquito-borne CHIKV infection causes a febrile illness (chikungunya fever) typically accompanied by rash and severe, debilitating arthralgia [42]. The CHIKV is considered a high priority emerging infection because of its mass dissemination potential, high morbidity and case-fatality rates, and major health impact [43]. So far, there are limited prospects for controlling CHIKV circulation and many parts of the Americas are now at high risk of major epidemics [44]. In this analysis, we looked at recent data available on the global research network on vaccines against the CHIKV, with particular interest in S&T organizations.

### Data processing

Data was collected from published and ‘in press’ articles retrieved from the Web of Science (WoS) database, covering the period 2010–2014. Queries were made in advanced search mode directed to the topic of research, which included title, abstract and keywords. The query was made using the terms "vaccin* AND (chikungunya OR CHIKV)". The search retrieved 129 papers.

Collected data was imported into the VantagePoint® software (Search Technology Inc.) using specific filters for the WoS database. Standardization of the various record names of an organization was done using the ‘list cleanup’ function of the software. Treated and cleaned data were formatted by VantagePoint® into an adjacency matrix to map co-authorships (co-occurrences). The matrix was converted into a csv (comma separated values) file and imported into the Gephi software [37] for visualization and analysis. In Gephi, the ForceAtlas 2 layout was used for visualization. Three different measures of centrality were calculated to assess whether an organization had a prominent or influential role in the network: degree centrality, betweeness centrality and closeness centrality.

Networks were created for both collaborating countries and research organizations using author affiliations to identify this information. Nodes represented countries or organizations, and links between them indicate that their members share the authorship of one or more scientific papers.

### Results

**Fig. 1:**
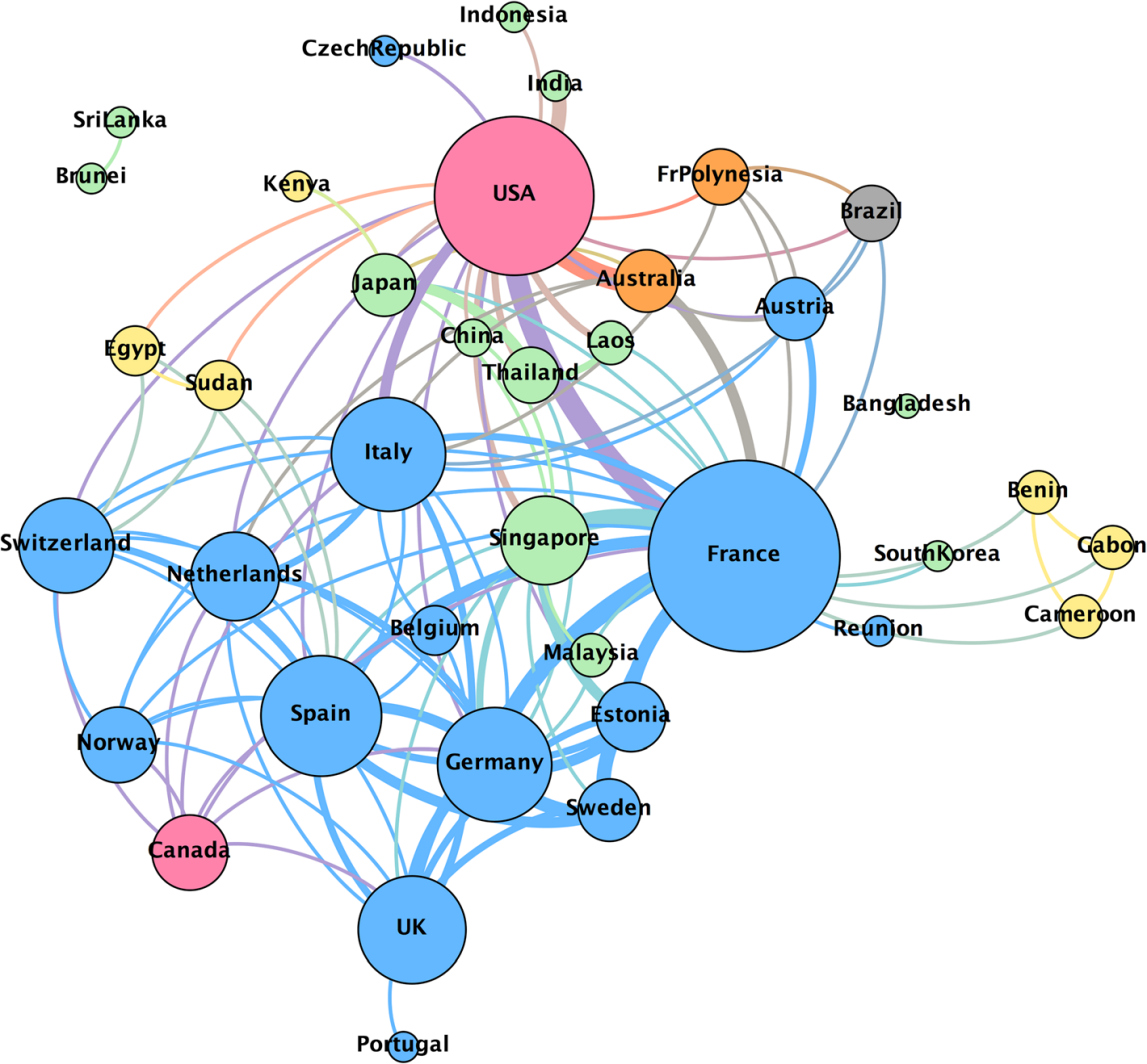
Global network of countries conducting research on vaccines against Chikungunya. Each node represents a country and two countries were considered connected if its organizations shared the authorship of a paper. The size of the nodes indicates their degree centrality and the thickness of links indicates the intensity of collaboration between two nodes. The nodes are color-coded by continent – European continent (*blue*), North America (*pink*), Asia (*green*), Africa (*yellow*), South America (*grey*) or Australia/Oceania (*orange*)

Based on the data retrieved, the global research network for the development of vaccines against CHIKV was composed of 38 countries, reflecting the solid international collaborative research efforts for disease control. The top three most central countries, according to their degree centrality, were France, Spain and the United States, as represented by the larger nodes (Fig. 1). During the 5-year period evaluated, these countries have collaborated with 26, 21 and 15 partner countries, respectively, and their degree centrality is indicated in Table 2. Degree centrality is a proxy for collaboration and not always a measure of the volume of publications. France, for example, had the highest degree centrality in the network but it was behind the United States in number of publications (Table 2). India and Singapore, despite publishing more papers than Spain on this specific subject (18 and 14 papers, respectively), ranked lower in degree centrality indicating a more nationally oriented research. The United States was the country that collaborated most with France, as indicated by the increased thickness of the links connecting them (Fig. 1).

**Table 2:** Degree centrality and number of publications in the global network of countries conducting research on vaccines against the Chikungunya virus

Country	Rank	Degree centrality^a^	Number of publications
France	1	0.702	22
United States	2	0.567	59
Spain	3	0.405	7

^a^Centrality values were normalized in accordance with the size of the network

The network of countries reflects the underlying research networks between organizations and research groups. Mapping of organizations involved in the global network for research on CHIKV vaccines identified 205 organizations. Academic organizations represent 49.3%, research institutes 22.9%, industry 10.7%, and hospitals and medical centres 7.3%. Other types of organizations, such as government-related institutions and funding agencies, were also present to a minor extent.

Metrics of the organizational network are shown in Table 3. The low density and centralization values associated with the large number of communities suggest sparse collaboration patterns among organizations and/or diverse research interests. These results highlight the need and opportunity for further development and strengthening of new collaborations.

**Table 3:** Metrics of the global organizational network of research on vaccines against the Chikungunya virus

Network metrics	Value
Number of nodes (organizations)	205
Number of links	493
Density	0.048
Centralization	0.128
Number of communities	40

**Fig. 2:**
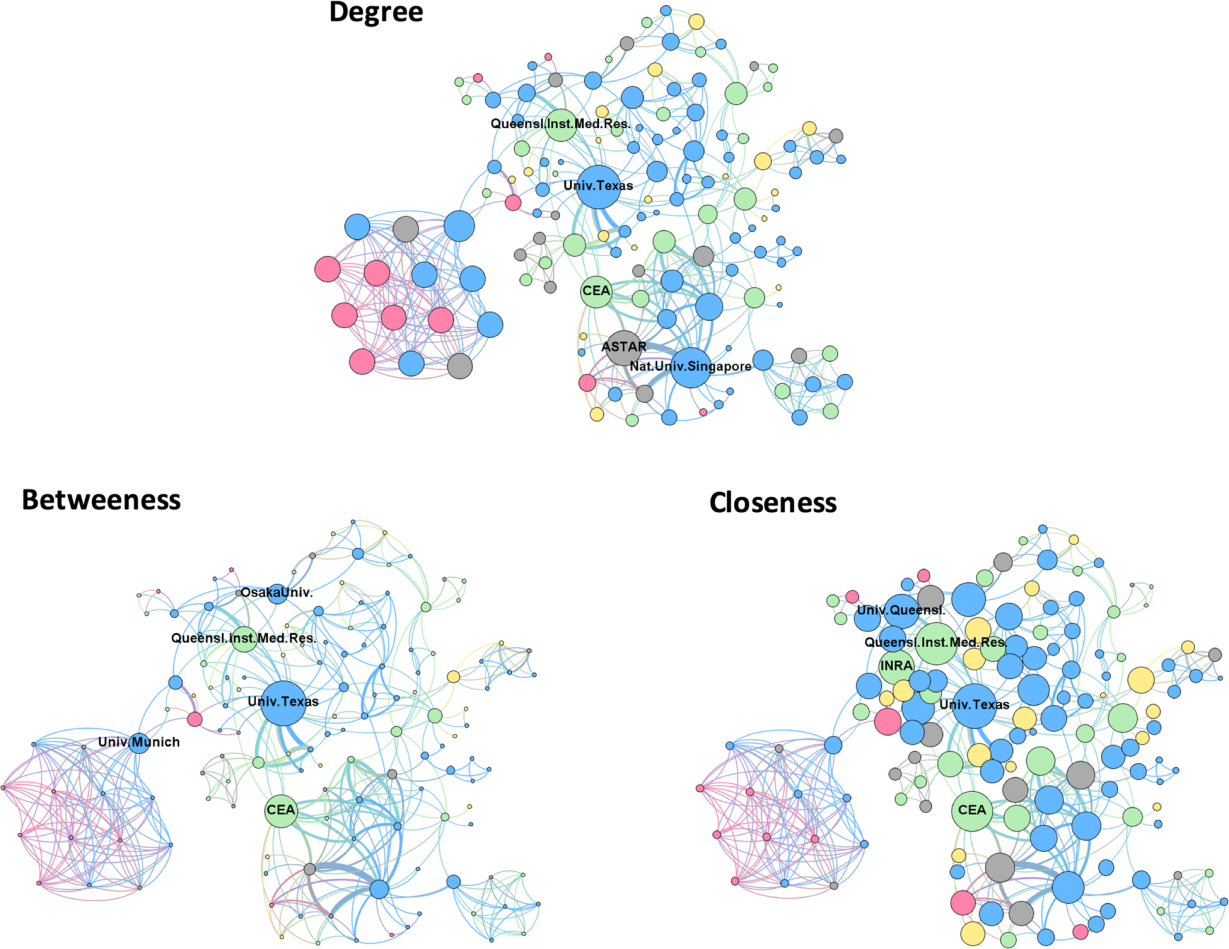
Global network of organizations that perform research on vaccines against the Chikungunya virus. Each node is an organization and two organizations were considered connected if its members shared the authorship of a paper. The thickness of links indicates the intensity of collaboration between two nodes and the size of the nodes indicates their degree centrality (*top image*), betweeness centrality (*lower left*) and closeness centrality (*lower right*). Node color indicates whether the organizations is a university (*pink*), research institute (*green*), industry (*yellow*), hospital or medical centre (*blue*) or other (*grey*). The top five organizations with highest centrality according to each metric are labelled. For visualization purposes, only the largest component is shown

According to their degree centrality, the most central organizations in the network are, in order of importance, the University of Texas (United States); the National University of Singapore (Singapore); the Singapore Agency for Science, Technology and Research (ASTAR; Singapore); the Queensland Institute of Medical Research (Australia) and the French Alternative Energies and Atomic Energy Commission (CEA; France) (Table 4). Among these central organizations, the CEA is the only one collaborating with all of the other four central institutions. The Queensland Institute of Medical Research has collaborations with the University of Texas and the University of Singapore cooperates with ASTAR (Fig. 2, top image).

**Table 4:** Top five central organizations in the global network of research on vaccines against the Chikungunya virus

Organization (rank)	Degree centrality^a^	Organization (rank)	Betweeness centrality^a^	Organization (rank)	Closeness centrality^a^
University of Texas	0.117	University of Texas	0.188	University of Texas	0.402
National University of Singapore	0.107	CEA	0.132	Queensland Institute of Medical Research	0.392
ASTAR	0.093	Queensland Institute of Medical Research	0.099	CEA	0.383
Queensland Institute of Medical Research	0.083	University of Munich	0.077	INRA	0.355
CEA	0.083	Osaka University	0.073	University of Queensland	0.354

^a^Centrality values were normalized in accordance with the size of the network

ASTAR, Singapore Agency for Science, Technology and Research; CEA, French Alternative Energies and Atomic Energy Commission; INRA, French National Institute for Agricultural Research

Some organizations that did not rank high on degree centrality can have central roles based on their betweeness or closeness measures (Table 4). The University of Munich (Germany) is a clear example of an organization that is acting as an intermediary (betweeness) linking two groups of institutions that would not otherwise be connected (Fig. 2, lower left). This gives it power and influence, as it is likely to control the information and knowledge flow between these two separate groups. In this network, values of closeness centrality are evenly distributed, indicating that most organizations can quickly obtain and disseminate information (Fig. 2, lower right).

### Discussion

The increasing circulation of CHIKV has promoted scientific development towards disease control. Although the CHIKV mostly affects African and Asian countries, there is a real possibility that it might reach regions of Europe and the Americas. Several factors influenced this global epidemic, including the increased volume of travellers, the widening geographic distribution of the mosquito vectors, the susceptibility of mosquitoes in non-endemic regions and the occurrence of autochthonous outbreaks [45].

Prior to 2004, the CHIKV was a relatively neglected and understudied pathogen, but it has been the focus of intense study in recent years [46, 47]. Even though the research and development of CHIKV vaccines began as early as the 1970s, interest and efforts waned until the recent re-emergence of the disease. Currently, no licensed vaccine against CHIKV is commercially available, but numerous candidates are under study.

In this analysis, the presence of France, the United States and Spain as most central in the research network reflects their scientific commitment with global health issues and the increasing trend of high-income countries to conduct research on diseases that used to be restricted to developing countries. After the 2005 and 2006 outbreaks on La Réunion, French researchers and institutes have published most CHIKV papers, but other countries are now paying close attention as well, as they, too, are at risk.

The participation of the industry in the organizational network (10.7%) for research on vaccines against the CHIKV reflects the economic interest in this area and highlights the influence of academic research in the industry, especially in the pharmaceutical setting. Similar levels of industry engagement in research networks has been reported in the area of infection and immunity [48]. As product development processes often rely on academic research to evolve and develop new ideas and techniques, networking can help companies to identify technological trends and potential partners for cooperation.

The University of Texas, top ranking in the three centrality measures evaluated, is one of the Centres of Excellence of the Global Virus Network, recently engaged in drug and vaccine development for CHIKV [49]. The University of Texas is also working on a live-attenuated vaccine with support from a large Japanese pharmaceutical company [47]. The presence of two S&T organizations from Singapore as central in this network could reflect the resurgence of the CHIKV experienced by the country in recent years [50]. ASTAR researchers together with CEA members are also part of the Integrated Chikungunya Research consortium network, which has recently developed a vaccine against the CHIKV already tested in a rodent model.

The analysis conducted in CHIKV vaccines revealed a good approximation of the research network structure and key players involved, which can help to indicate directions for further investigation. The relatively small and young research community is likely to evolve to a more stable network configuration in the following years, opening the potential for further in-depth analysis of the organizations and researchers involved. The analysis of different time intervals could provide insights on the dynamics of the research network. The integration of other data sources can add a more extensive perspective on the vaccine development community.

Currently, there is an emerging trend to use SNA for research policy recommendations [9, 10, 27]. Although there is still limited evidence regarding the effectiveness of SNA-based policies, the results presented herein inform policymakers on several relevant issues: (1) there is a global network fragmentation, indicating a need for consolidating collaboration; (2) the most central countries are high-income economies, revealing the need for their collaboration with low- and middle-income countries for research capacity strengthening; and (3) the identification of central organizations, which can act as sources of information on technology trends, is relevant to identify potential partners for global alliances and support strategic decisions on public health investments.

More general policy-relevant questions related to the results are open for discussion: To what extent should organizations focus their research efforts on one or two partners as opposed to collaborating with several different ones? How do local research settings influence international collaboration? How to reinforce collaboration between universities and industries? Who are the central researchers most likely to sustain networking and assist in guiding research policies?

## Conclusions

Co-authorship network analysis is a powerful method of retrieving information for health studies. This method contributes in innovative ways to the evaluation of the collaborative behaviour of researchers, organizations and countries, by revealing the cooperation structure combined with information about the centrality of the network participants. The multitude of applications demonstrates the versatility of information that can be recovered with this method and opens new perspectives in the study of collaboration. It allows to understand the research structure on specific topics, the evolution of research networks over time and the participation of a particular organization or country in a specific network.

Some limitations of this research method have to be mentioned. The co-authorship data represent only one of the possible indicators of scientific collaboration. Not all collaborative efforts result in publications, and not all co-authored papers necessarily imply collaboration in the form of knowledge sharing among authors. Still, it is assumed that, in most cases, co-authorship indicates an active cooperation between partners beyond the simple exchange of material or information.

## Abbreviations

ASTAR: Singapore Agency for Science, Technology and Research
CEA: French Alternative Energies and Atomic Energy Commission
CHIKV: Chikungunya virus
S&T: Science and technology
SNA: Social network analysis
